# Pembrolizumab-Associated Seronegative Myasthenia Gravis in a Patient With Metastatic Renal Cell Carcinoma

**DOI:** 10.7759/cureus.15174

**Published:** 2021-05-22

**Authors:** Shaunak K Pandya, Matthew Ulrickson, JiaXi Dong, Ryan Willen, Arati Pandya

**Affiliations:** 1 Internal Medicine, Banner - University Medical Center Phoenix, University of Arizona College of Medicine - Phoenix, Phoenix, USA; 2 Hematology and Oncology, Stem Cell Transplant, Banner MD Anderson Cancer Center, Gilbert, USA

**Keywords:** myasthenia gravis, pembrolizumab, checkpoint inhibitors, autoimmune, seronegative, renal cell carcinoma (rcc), intravenous immunoglobulin (ivig)

## Abstract

Seronegative myasthenia gravis is a rare, but potential adverse effect of immune checkpoint inhibition. There have been few but increasing number of cases reported in recent years, and early recognition is important for prompt diagnosis and management. Here, we describe the case of a 65-year-old male with metastatic renal cell carcinoma on pembrolizumab diagnosed with new-onset seronegative myasthenia gravis and review literature on its management.

## Introduction

Programmed cell death protein-1 (PD-1) is an inhibitory cell-surface receptor on T-lymphocytes that binds the ligands PD-L1 and PD-L2 [1]. PD-L1 and PD-L2 are expressed on the surface of T and B cells, dendritic cells, macrophages, and also on non-hematopoietic tissues, such as lung, endothelial, pancreatic islet, neurons, and keratinocytes [2]. A variety of malignancies have been found to express PD-L1 including non-small-cell lung cancer, ovarian, breast, cervical, colon, pancreatic, melanoma, gastric, and lymphoma. When PD-L1 on the surface of a tumor cell binds to PD-1, T-cell immune responses are downregulated, which enables survival of the malignant clone. Therefore, disruption of this binding releases the inhibition on T-cell activity, augmenting antitumor immune responses and clinical control of the tumor. This is the mechanism of action of checkpoint inhibitors, such as pembrolizumab and nivolumab, monoclonal antibodies that block signaling through the programmed death (PD-1) pathway for the treatment of various cancers [3]. With increasing use of these agents, there has been a growing number of reported patients diagnosed with myasthenia gravis (MG) while on checkpoint inhibitors, some with mortality rates as high as 30% [4]. Associated checkpoint inhibitors include pembrolizumab, nivolumab, and ipilimumab. To date, there have been few reported cases of pembrolizumab-induced MG, with manifestations including ocular MG, myopathy, and respiratory distress requiring mechanical ventilation [5-9]. Here, we describe a case of pembrolizumab-induced seronegative MG in a patient with metastatic renal cell carcinoma.

## Case presentation

A 65-year-old male with type 2 diabetes mellitus and hypothyroidism was initially diagnosed with clear cell renal carcinoma five years prior and was initially treated with left nephrectomy, radiation therapy, axitinib, and pazopanib. Due to refractory metastatic lesions involving lung and bone, patient was started on pembrolizumab. After his third infusion of pembrolizumab, he developed bilateral ptosis, diplopia, and dyspnea. Initially, the visual changes were attributed to macular edema/thickening on ophthalmology evaluation. Due to persistent symptoms, he was empirically started on prednisone 60 mg daily at an outside facility with some improvement in ocular symptoms. Pembrolizumab was discontinued, although there was favorable response since its initiation with marked reduction in size and number of lung metastases.

Two months later, the patient was admitted to our facility for worsening dyspnea, diplopia, and bilateral ptosis despite ongoing prednisone. On admission, patient was hemodynamically stable with a negative inspiratory force of -60 cmH­_2_O. He was found to have bilateral ptosis, difficulty maintaining upward gaze, and incremental bilateral upper extremity weakness on repetitive testing, consistent with MG. Recent CT chest demonstrated a 5-mm pleural-based nodule in left lower lobe, but no thymoma, and no further imaging was obtained. Serology testing for MG was negative for muscle-specific kinase (MuSK) antibody, striated muscle antibody, and acetylcholine receptor (AChR) antibodies including binding, blocking, and modulating antibody panel. Voltage-gated calcium channel type P/Q antibody was also negative.

He was started on intravenous immunoglobulin (IVIG) 2 g/kg over a five-day course, prednisone 50 mg daily, and pyridostigmine as part of his treatment guided by neurology. Pembrolizumab remained held since the initial onset of symptoms. His symptoms improved with the IVIG course and he was subsequently transitioned to pyridostigmine 60 mg three times daily to maintain response. His respiratory status remained stable on 2-4 liters supplemental oxygen via nasal cannula throughout his hospital stay. Patient tolerated IVIG, steroids, and pyridostigmine without adverse effects and he was discharged on pyridostigmine with outpatient neuromuscular clinic follow-up for electromyography and nerve conduction testing.

## Discussion

MG is an autoimmune phenomenon characterized by fluctuating muscle weakness involving ocular, bulbar, respiratory, and limb muscles. Pathogenesis involves antibodies affecting the interaction between presynaptic nerve endings and postsynaptic muscle fibers at the neuromuscular junction (NMJ), but seronegative cases also occur. Serological testing is largely focused on the presence of antibodies against the AChRs and MuSK. Up to 15% of patients do not have detectable AChR antibodies, of which about 40% have detectable MuSK antibodies [10]. Seronegative MG occurs in a small fraction of patients who lack any detectable AChR or MuSK antibodies.

Treatment of seronegative MG is similar to other subtypes, involving symptomatic treatment with acetylcholinesterase inhibitors (such as oral pyridostigmine), immunosuppression with glucocorticoids or azathioprine, and immunomodulatory interventions with IVIG or plasmapheresis [11,12]. Thymectomy is a treatment option in select patients, especially with thymoma-associated MG. Pyridostigmine improves acetylcholine interaction with its receptor in the synaptic cleft, thereby improving symptoms. It is often started at 60 mg orally three times a day with increases of 30-60 mg until symptoms improve. Cholinergic side effects involving gastrointestinal tract, sweating, and bradycardia are possible, especially when dose increases above 300 mg/day. Glucocorticoids, such as prednisone, are used with two approaches: slow induction with low-dose prednisone (10 mg orally/day with slow up-titration by 10 mg every 5-7 days) in milder cases or quick induction with high-dose prednisone (1-1.5 mg/kg/day), which can result in worsening symptoms in the initial days of treatment. Other immunosuppressant agents used less frequently include azathioprine, mycophenolate mofetil, cyclosporine, and methotrexate. In patients with symptomatic MG not responding to immunosuppression or severe MG with impending/ongoing myasthenia crisis, additional treatment is necessary with IVIG (2 g/kg over 3-5 days) or plasmapheresis.

In this case, patient was symptomatic despite ongoing treatment with high-dose steroids, which prompted additional treatment with IVIG. Given the patient's clinical presentation consistent with NMJ involvement and lack of central nervous system (CNS) or distal peripheral nerve involvement, further serologic assessment beyond the NMJ for concurrent paraneoplastic syndrome was deferred. Paraneoplastic neurologic syndromes have been associated with various onconeural antibodies resulting from cross-reactivity with tumor [13]. They can target the CNS (limbic encephalitis, cerebellar ataxia), peripheral nervous system (sensory neuropathy), or NMJ (Lambert-Eaton syndrome, MG). Though MG can independently manifest as a paraneoplastic syndrome, the association with de novo diagnosis after starting pembrolizumab suggests a likely checkpoint-inhibitor-associated presentation of MG.

PD-1 inhibitors have been an important addition to treatment options in the field of oncology, but they are associated with various immune-related adverse effects including neurologic, gastrointestinal, pulmonary, endocrine, and other organ systems [14]. PD-1 modulates against autoreactivity, and its inhibition may lead to increased autoantibody production. This mechanism may be similar to other checkpoint inhibitors (CTLA-4), which have shown enhanced T-cell response and increased anti-AChR antibody production [15]. No clear clinical risk factors have been identified for PD-1 inhibitor-associated MG. However, cancer patients receiving checkpoint inhibitor therapy with pre-existing autoimmune diseases have been shown to have exacerbations or immune-related adverse event rates as high as 75% [16]. In 2017, Makarious and colleagues reported 23 cases of checkpoint-inhibitor-associated MG, of which 72.7% were de novo with no prior history; remaining were exacerbations [4]. MG-related mortality was reported to be as high as 30%. This highlights the importance of recognizing early manifestations of MG, frequent respiratory assessments, and collaborative management with neurology as the use of checkpoint inhibitors continues to grow in oncology.

## Conclusions

In the setting of PD-1 inhibitory treatment, MG can be life-threatening. Early recognition through detailed history and physical examination should prompt early serologic testing for diagnosis. Prompt treatment, including discontinuation of the PD-1 inhibitor, steroids, acetylcholinesterase inhibitors, and IVIG, may be lifesaving. Management of PD-1 inhibitor-related MG requires close collaboration with neurology and oncology team members.
